# China collaborative study on dialysis: a multi-centers cohort study on cardiovascular diseases in patients on maintenance dialysis

**DOI:** 10.1186/1471-2369-13-94

**Published:** 2012-08-30

**Authors:** Fanfan Hou, Jianping Jiang, Jianghua Chen, Xueqing Yu, Qiugen Zhou, Pingyan Chen, Changlin Mei, Fei Xiong, Wei Shi, Wei Zhou, Xusheng Liu, Shiren Sun, Di Xie, Jun Liu, Ping Zhang, Xiao Yang, Yixiang Zhang, Yanmin Zhang, Xinling Liang, Zhimin Zhang, Qizhan Lin, Yan Yu, Shengjie Wu, Xin Xu

**Affiliations:** 1 Division of Nephrology, Nanfang Hospital, Southern Medical University, 1838 North Guangzhou Avenue, Guangzhou 510515, China; 2 Key Lab for Organ Failure Research, Ministry of Education, Guangzhou, China; 3 Division of Nephrology, The First Affiliated Hospital, Medical College of Zhejiang University, Hangzhou, China; 4 Division of Nephrology, The First Affiliated Hospital, Sun Yat-Sen University, Guangzhou, China; 5 Department of Biostatistics, Southern Medical University, Guangzhou, China; 6 Division of Nephrology, Changzheng Hospital, Secondary Military Medical University, Shanghai, China; 7 Division of Nephrology, The First People’s Hospital, Wuhan, China; 8 Division of Nephrology, Guangdong General Hospital, Guangzhou, China; 9 Division of Nephrology, The 309th Hospital of People’s Liberation Army, Beijing, China; 10 Division of Nephrology, Guangdong Provincial Hospital of Traditional Chinese Medicine, Guangzhou, China; 11 Division of Nephrology, Xijing Hospital, Fourth Military Medical University, Xi’an, China

**Keywords:** Cardiovascular morbidity, Dialysis modality, Risk factor

## Abstract

**Background:**

Cardiovascular disease (CVD) is the main cause of death in patients on chronic dialysis. The question whether dialysis modality impacts cardiovascular risk remains to be addressed. China Collaborative Study on Dialysis, a multi-centers cohort study, was performed to evaluate cardiovascular morbidity during maintenance hemodialysis (HD) and peritoneal dialysis (PD).

**Method:**

The cohort consisted of chronic dialysis patients from the database of 9 of the largest dialysis facilities around China. The inclusion period was between January 1, 2005, and December 1, 2010. Cardiovascular morbidity was defined as the presence of clinically diagnosed ischemic heart disease, heart failure, peripheral vascular disease, and/or stroke. The patients who had cardiovascular morbidity before initiation of dialysis were excluded. Data collection was based on review of medical record.

**Result:**

A total of 2,388 adult patients (1,775 on HD and 613 on PD) were enrolled. Cardiovascular morbidity affected 57% patients and was comparable between HD and PD patients. However, clinically diagnosed ischemic heart disease and stroke was more prevalent in PD than HD patients. When the patients were stratified by age or dialysis vintage, the cardiovascular morbidity was significantly higher in PD than HD among those aged 50 years or older, or those receiving dialysis over 36 months. Multivariate analysis revealed that the risk factors for cardiovascular morbidity had different pattern in PD and HD patients. Hyperglycemia was the strongest risk factor for cardiovascular morbidity in PD, but not in HD patients. Hypertriglyceridemia and hypoalbuminemia were independently associated with CVD only in PD patients.

**Conclusions:**

Cardiovascular morbidity during chronic dialysis was more prevalent in PD than HD patients among those with old age and long-term dialysis. Metabolic disturbance-related risk factors were independently associated with CVD only in PD patients. Better understanding the impact of dialysis modality on CVD would be an important step for prevention and treatment.

## Background

Cardiovascular disease (CVD) is the leading cause of death among patients undergoing maintenance dialysis. Compared with the general population, dialysis patients have a 10 to 20 times greater incidence of cardiovascular death [1-4]. The need to identify CVD and its risk factors in such population has never been greater.

Difference in the burden of cardiovascular morbidity in dialysis patients among selected countries has been described previously [5]. The prevalence of coronary artery disease in Asian dialysis population seems relatively lower than that in patients from western countries [5-7]. The reason for the difference has not been clarified but may correlate to the background cardiovascular morbidity in their respective general population [8], as well as the baseline characteristics of the dialysis population.

China is a country with huge population and increasing burden of ESRD [9]. Our and others’ previous studies [6,7,9] have showed the characteristics of Chinese dialysis population. Compared with the United States and Europe where diabetes was the number one cause of ESRD, the leading cause of ESRD in China is chronic glomerulonephritis [6,7,9]. The average age of Chinese dialysis patients was ten-years younger than those in western countries [6,7]. It is reasonable to hypothesize that the pattern of CVD and the risk factors for CVD in Chinese dialysis patients might be different from that in other ethnic populations. However, despite the large and growing population of maintenance dialysis in China, there has been lack of data on CVD in this population.

Risk factors for CVD in dialysis patients include those that affect the general population (traditional risk factors) and those related to end stage renal disease (ESRD) ( non-traditional risk factors), as well as those that are specific to chronic dialysis. Although the faster deterioration of CVD in ESRD patients has been considered due mainly to accumulation of traditional and non-traditional risk factors [10-12], the question whether dialysis modality impacts cardiovascular risk remains to be addressed. Cardiovascular disease is often present in patients initiating dialysis, but may also develop during chronic dialysis treatment. To address the relevant question, it is important to know the impact of dialysis modality on outcome in patients with existing CVD, and also, on emergence of “de novo” CVD during dialysis. However, there are few studies comparing CV risk of peritoneal dialysis (PD) versus hemodialysis (HD) patients and evaluating the impact of dialysis on development of CVD. Outlining the epidemiological data on CVD in patients on HD and PD might be an important step to evaluate the impact of dialysis modality on CVD.

The goal of the present study was to evaluate the prevalence of CVD in a representative Chinese population undergoing maintenance dialysis. We also compared the prevalence and risk factors of CVD between patients on HD and PD.

## Results and discussion

### Patient characteristics in the CCSD cohort

A total of 2,388 adult patients were enrolled in CCSD. Both maintenance HD (n = 1,775) and PD (n = 613) patients were included. The patient characteristics were showed in Table 1. In this cohort, 1313 (55%) were male. The mean age was 54 years old. The median time for dialysis was 26 months (12–51 months). In regard to the etiology of ESRD, primary glomerular disease was the most frequent, affecting 1097 (45.9%) patients. Diabetes was the second (21.4%). In this representative cohort, the predominant patients (75%) were on HD and 25% patients were on PD. Compared with HD patients, PD patients were younger with shorter dialysis vintage. In addition, hypertension, elevated BMI, and hypertriglyceridemia were more prevalent in PD patients.

**Table 1:** **Characteristics of the Chinese dialysis patients**^**a**^

	**Total**	**Dialysis modality**		
	**(n=2388)**	**PD (n=613)**	**HD (n=1775)**	***P***
**Demographic**				
Age, yr	54.1±15.3	51.0±15.0	55.1±15.3	<0.01
Male, n (%)	1313 (55.0 )	309 (50.4)	1004 (56.6)	<0.01
Smoker, n (%)	117 (4.9)	29 (4.7)	88 (5.0)	0.91
Diabetes, n (%)	548 (22.9)	136 (22.2)	412 (23.2)	0.62
Hypertension, n (%)	2013 (84.3)	566 (92.3)	1447 (81.5)	<0.01
**Dialysis vintage, mo**	26 (12-51)	19 (9-35)	30 (13-60)	<0.01
**Dialysis adequacy, kt/v**^**b**^		2.1±0.5	1.6±0.4	NA
**Physical examination**				
Body mass index, kg/m^2^	21.7±3.4	22.1±3.2	21.5±3.4	<0.01
Systolic BP, mmHg	143.1±22.4	141.6±23.2	143.6±22.0	0.05
Diastolic BP, mmHg	83.4±13.1	83.1±13.1	83.5±13.1	0.57
**Cause of renal failure, n (%)**				
Diabetes	512 (21.4)	129 (21.0)	383 (21.6)	0.82
Hypertension	406 (17.0)	113 (18.4)	293 (16.5)	0.29
Glomerular disease	1097 (45.9)	323 (52.7)	774 (43.6)	<0.01
Tubulointerstitial disease	77 (3.2)	31 (5.1)	46 (2.6)	<0.01
Connective tissue disease	48 (2.0)	11 (1.8)	37 (2.1)	0.74
Polycystic kidney disease	116 (4.9)	17 (2.8)	99 (5.6)	<0.01
Others unknown causes	316 (13.2)	39 (6.4)	277 (15.6)	<0.01
**Laboratory assessments**				
Albumin, g/L	39.5±6.0	39.0±5.5	40.0±6.1	<0.01
Hemoglobin, g/L	104.3±20.5	106.8±20.8	103.5±20.3	<0.01
C-reactive protein, mg/L	3.6 (1.9-9.4)	3.1 (1.3-8.6)	3.9 (2.1-10.0)	<0.01
Glucose, mM	4.8 (4.3-5.6)	4.8 (4.3-5.6)	4.9 (4.3-5.6)	0.33
Triglyceride, mM	1.4 (1.0-2.1)	1.5 (1.1-2.4)	1.3 (1.0-2.0)	<0.01
LDL cholesterol, mM	2.4±0.8	2.8±0.8	2.3±0.7	<0.01
HDL cholesterol, mM	1.1±0.3	1.1±0.3	1.1±0.4	0.21
Serum phosphate, mM	2.0±0.5	2.0±0.5	2.0±0.6	0.18
Serum corrected calcium, Mm	2.2±0.2	2.3±0.2	2.2±0.2	0.17
iPTH, pg/ml	278.0 (138.5-572.4)	333.6 (171. 5-589.2)	260.1 (128.9-564.9)	0.72
Ferritin, ng/ml	233.9 (97.3-513.1)	157.5 (68.9-317.8)	284.1 (115.1-593.0)	<0.01
**Medication, n (%)**				
ACE inhibitors / ARBs	1217 (51.0)	363 (59.2)	854 (48.1)	<0.01
β-blockers	1106 (46.3)	342 (55.8)	764 (43.0)	<0.01
calcium channel blockers	1672 (70.0)	475 (77.5)	1197 (67.4)	<0.01
erythropoietin	2036 (85.3)	546 (89.1)	1490 (83.9)	<0.01
calcitriol	1004 (42.0)	252 (41.1)	752 (42.4)	0.60
phosphate-binding agents	967 (40.5)	255 (41.6)	712 (40.1)	0.54
lipid-lowering drugs	269 (11.3)	118 (19.2)	151 (8.5)	<0.01
Aspirin	180 (7.5)	67 (10.9)	113 (6.4)	<0.01

**a**: Continuous variables were expressed as mean ± SD for normally distributed data, median (25th percentile -75th percentile) for skewed data, while categorical variables were expressed as percentage (%).

**b**: kt/v is weekly in PD and per session in HD.

**Abbreviation:** HD, hemodialysis; PD, peritoneal dialysis; BP, blood pressure; iPTH: intact parathyroid hormone; ACE inhibitors: angiotensin-converting enzyme inhibitor; ARBs, angiotensin receptor blockers; NA, not applicable.

### Cardiovascular morbidity in the CCSD cohort

Cardiovascular morbidity was defined as any description of clinically diagnosed ischemic heart disease, heart failure, stroke, or peripheral vascular disease in each medical record from initiation of dialysis. As shown in Table 2, cardiovascular morbidity affected 57% patients in this cohort. Even though the overall cardiovascular morbidity was comparable between HD and PD patients (56.3% *vs.* 58.9%), the proportion of patients with ischemic heart disease and stroke was significantly more prevalent in patients on PD than those on HD (Table 2).

**Table 2:** The cardiovascular morbidity in Chinese dialysis patients

	**Total**	**Dialysis modality**		
	**(n=2388)**	**PD (n=613)**	**HD (n=1775)**	***P***
Cardiovascular morbidity, n (%)^a^	1361 (57.0)	361 (58.9)	1000 (56.3)	0.28
IHD	543 (22.7)	170 (27.7)	373 (21.0)	<0.01
HF	1051 (44.0)	290 (47.3)	761 (42.9)	0.06
PVD	78 (3.3)	23 (3.8)	55 (3.1)	0.43
Stroke	230 (9.6)	82 (13.4)	148 (8.3)	<0.01

**a:** Defined as any description of ischemic heart disease (IHD), heart failure (HF), stroke or peripheral vascular disease (PVD).

**Abbreviation:** HD, hemodialysis; PD, peritoneal dialysis.

**Figure 1:**
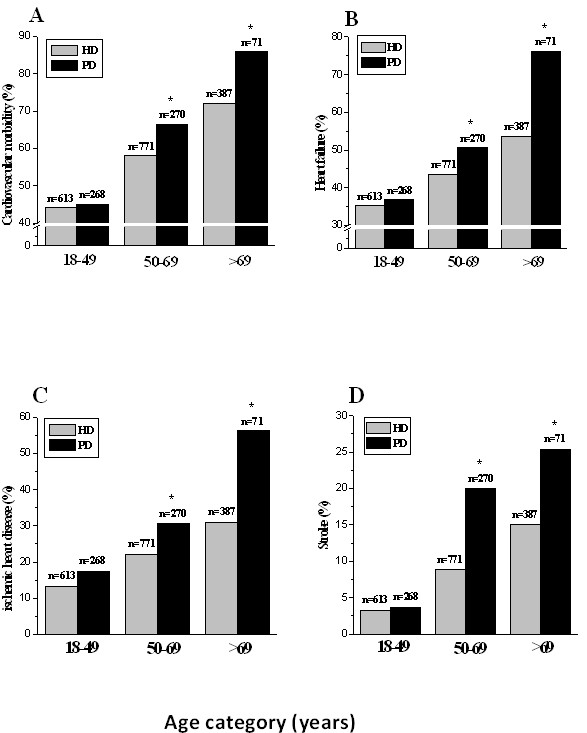
**Cardiovascular morbidity (A), heart failure (B), coronary heart disease (C) and the stroke (D) in each category of age among HD and PD patients.** *P < 0.05 *vs*. HD patients.

**Figure 2:**
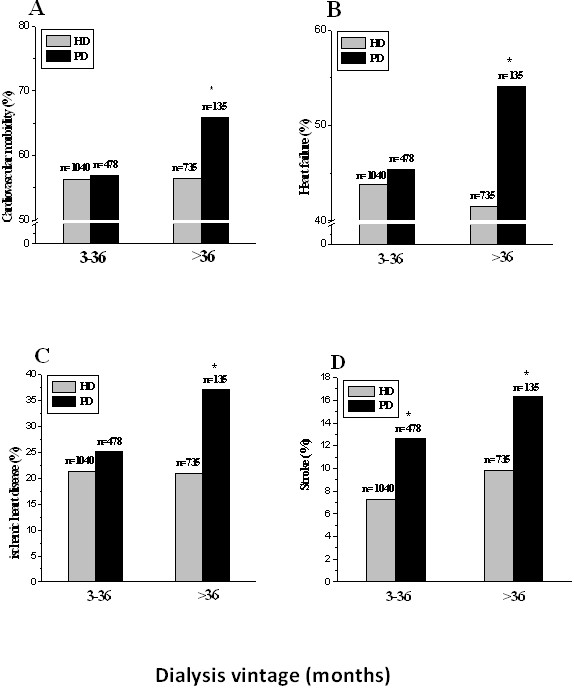
**Cardiovascular morbidity (A), heart failure (B), coronary heart disease (C) and the stroke (D) in each category of dialysis vintage among HD and PD patients.** *P < 0.05 *vs*. HD patients

Since development of CVD is a time-dependent process and there was a difference in age and dialysis vintage between HD and PD population, we further compared the cardiovascular morbidity in patients stratified by age and dialysis vintage, separately. As shown in Figure 1A, the cardiovascular morbidity was significantly more prevalent in PD than HD among those aged 50 years or older. The similar trend was observed in clinically diagnosed heart failure, ischemic heart disease, and stroke (Figure 1 B-D). When comparison was made in patients stratified by dialysis vintage, the overall cardiovascular morbidity, heart failure, and ischemic heart disease were also more prevalent in PD than HD patients among those receiving dialysis for more than 36 months (Figure 2A-C). The proportion of stroke was significantly higher in PD compared to HD patients in each category of dialysis vintage (Figure 2D). Since the number of patients with peripheral artery disease was limited, analysis according to stratification was not performed.

In our cohort, the mean age of HD patients was higher than that of PD population. Higher mortality in older patients may result in lower cardiovascular morbidity in survivors. To reduce the bias, we compared mortality during the evaluation period between HD and PD patients. The annual rate of all-cause mortality was 8% in HD and 10% in PD patients. The proportion of cardiovascular death in total mortality was comparable between HD and PD population (52% *vs.* 48%).

### Risk factors of CVD in the CCSD cohort

To identify the risk factors correlated with CVD, we conducted a univariate and a multivariable logistic regression analysis. Variables associated with CVD at the univariate analysis were shown in Table 3. Odds ratios were obtained from logistic regression analysis. The risk factors that were significant at *P* < 0.05 were then simultaneously entered into the stepwise logistic regression to identify independent risk factors for CVD. As shown in Table 4, the risk factors for cardiovascular morbidity in this cohort included old age, smoking, hypertension, Hypertriglyceridemia, low level of hemoglobin, and elevated levels of C-reactive protein. It is interesting to note that the risk factors of CVD showed different pattern in PD and HD patients. Hyperglycemia was the strongest risk factor for CVD in PD (OR, 2.43; 95% CI, 1.22-2.62), but not in HD patients. Hypertriglyceridemia and hypoalbuminemia were the independent risk factors for CVD only in PD population (Table 4).

**Table 3:** Univariable logistic regression analysis: variables related to cardiovascular morbidity in dialysis patients

**Variables**	**Total (n=2388)**		**PD (n=613)**		**HD (n=1775)**	
	**OR (95% CI)**	***P***	**OR (95%CI)**	***P***	**OR (95%CI)**	***P***
Age ≥50 yr, (no=0, yes=1)	2.27 (1.91-2.69)	<0.01	2.89 (2.07-4.03)	<0.01	2.13 (1.75-2.60)	<0.01
Gender (F=0, M=1)	1.07 (0.91-1.26)	0.39	1.00 (0.72-1.38)	1.00	1.09 (0.91-1.32)	0.35
Smoking (no=0, yes=1)	2.38 (1.55-3.65)	<0.01	2.27 (0.96-5.40)	0.06	2.42 (1.48-3.96)	<0.01
Hypertension (no=0, yes=1)	1.91 (1.53-2.39)	<0.01	2.48 (1.34-4.57)	<0.01	1.82 (1.43-2.32)	<0.01
BMI ≥24.0kg/m^2^, (no=0, yes=1)	1.21 (1.00-1.47)	0.05	1.52 (1.04-2.22)	0.03	1.11 (0.88-1.39)	0.39
Glucose >7.0mM, (no=0, yes=1)	2.35 (1.73-3.21)	<0.01	4.19 (2.21-7.98)	<0.01	1.88 (1.31-2.69)	<0.01
Triglyceride >1.7mM, (no=0, yes=1)	1.23 (1.02-1.47)	0.03	1.85 (1.31-2.60)	<0.01	1.02 (0.82-1.28)	0.83
LDL cholesterol >3.36mM, (no=0, yes=1)	0.98 (0.74-1.29)	0.88	0.68 (0.46-1.02)	0.06	1.50 (0.49-2.27)	0.06
HDL cholesterol ≤0.92mM, (no=0, yes=1)	1.06 (0.87-1.30)	0.55	1.35 (0.92-1.97)	0.13	0.97 (0.76-1.23)	0.79
Albumin ≤35g/L, (no=0, yes=1)	1.36 (1.11-1.66)	<0.01	2.18 (1.41-3.36)	<0.01	1.18 (0.93-1.48)	0.17
Hemoglobin ≤90g/L, (no=0, yes=1)	1.33 (1.10-1.61)	<0.01	0.82 (0.55-1.23)	0.34	1.54 (1.23-1.93)	<0.01
C-reactive protein >3mg/L, (no=0, yes=1)	1.53 (1.25-1.87)	<0.01	1.70 (1.20-2.41)	<0.01	1.44 (1.13-1.84)	<0.01
Ferritin, ng/ml	1.00 (1.00-1.00)	0.06	1.00 (0.99-1.00)	0.86	1.00 (1.00-1.00)	0.04
Dialysis vintage (mo)	1.00 (1.000-1.00)	0.15	1.01 (1.00-1.02)	0.02	1.00 (1.00-1.00)	0.05

**Abbreviation:** HD, hemodialysis; PD, peritoneal dialysis; BMI, body mass index; OR, odds ratio; CI, confidence interval.

**Table 4:** Multivariable logistic regression analysis: variables related to cardiovascular morbidity in dialysis patients

**Variables**	**Total (n=2388)**		**PD (n=613)**		**HD (n=1775)**	
	**OR (95% CI)**	***P***	**OR (95%CI)**	***P***	**OR (95%CI)**	***P***
Age ≥50 yr, (no=0, yes=1)	2.31 (1.83-2.93)	<0.01	2.27 (1.53-3.37)	<0.01	2.45 (1.83-3.82)	<0.01
Smoking (no=0, yes=1)	1.831 (1.26-2.67)	<0.01			1.74 (1.18-2.56)	<0.01
Hypertension (no=0, yes=1)	2.19 (1.57-3.05)	<0.01	2.38 (1.12-5.03)	0.02	2.19 (1.51-3.15)	<0.01
Glucose >7.0mM, (no=0, yes=1)			2.43 (1.22-2.62)	0.01		
Triglyceride >1.7mM, (no=0, yes=1)	1.27 (1.00-1.62)	<0.05	1.77 (1.19-2.62)	<0.01		
Albumin ≤35g/L, (no=0, yes=1)			1.87 (1.08-3.23)	0.03		
Hemoglobin ≤90g/L, (no=0, yes=1)	1.43 (1.07-1.91)	0.02			1.64 (1.16-2.32)	<0.01
C-reactive protein >3mg/L, (no=0, yes=1)	1.33 (1.05-1.68)	0.02			1.45 (1.09-1.92)	0.02

**Abbreviation:** HD, hemodialysis; PD, peritoneal dialysis; OR, odds ratio; CI, confidence interval.

## Discussion

We found in the present study that cardiovascular morbidity prevalence remains as high as 57% in Chinese dialysis population, even with less diabetes and more glomerulonephritis comparing to Western countries. Compared to HD patients, PD patients had more prevalent CVD among those aged 50 years or older and those receiving dialysis for more than 36 months. The risk factors for cardiovascular morbidity in PD patients had different pattern compared to HD population.

The question whether dialysis modality impacts the risk of CVD remains to be addressed. It is important to know the impact of dialysis modality not only on outcome in patients with existing CVD, but also on emergence of “de novo” CVD during dialysis. However, there are few studies evaluating the development of CVD in dialysis patients. Our study found that clinically diagnosed CVD after initiation of dialysis was more prevalent than expected. The ischemic heart disease was 22.7%, heart failure 44%, stroke 9.6%, and peripheral artery disease 3.3%. Heart failure was the most prevalent cardiovascular morbidity occurred during chronic dialysis.

Studies comparing the outcomes with PD and HD patients have shown conflicting results [13-15], probably due to difference in study populations (incident or prevalent dialysis patients) or methodology. Therefore, valid comparison between HD and PD requires patient stratification according to the major risk factors [16]. The present study showed that the overall cardiovascular morbidity was comparable between HD and PD patients. However, this result could be biased by the difference in age and dialysis vintage between patients on the two dialysis modality since CVD is a time-dependent process. To reduce such bias, we classified the patients by age and dialysis vintage. We found that cardiovascular morbidity and its composites such as heart failure, ischemic heart disease, and stroke were significantly higher in PD compared to HD patients among those aged 50 years or older and those receiving dialysis for more than 3 years. The lower cardiovascular morbidity in HD patients was not related with high mortality since annual rate of all-cause and cardiovascular mortality was relatively lower in patients on HD.

 The reasons for the increased cardiovascular morbidity in our PD patients remained elusive. There might be two explanations. First, the PD patients enrolled in the study were all treated by the conventional PD solution containing high concentration of glucose, since icodextrin- or amino acid-based dialysate is not commercially available in China. Long-term exposure to high level of glucose or glucose degradation products may lead to cumulative metabolic stress and resulting in development or worsening of CVD [17,18]. This hypothesis was supported by the fact that hyperglycemia served as the strongest independent risk factor in the multiple logistic regression model than the other known risk factors. Furthermore, increased BMI and hypertriglyceridemia were more common in PD than HD patients. Alternatively, the prevalence of hypertension, probably reflecting an inadequate control of fluid volume, was more common in PD than HD patients in our population (92.3% *vs*. 81.5%).

The risk factors for CVD specifically related to chronic dialysis have not been well established. Traditional and non-traditional CVD risk factors were both identified in our cohort. However, the prevalence of traditional risk factors such as diabetes, smoking, higher BMI, and hyperlipidemia was less frequent as compared with dialysis population in western countries [5,19]. Meanwhile, uremia-related risk factors seemed more prevalent in our cohort. Even though erythropoietin was widely used (85%), anemia was very common in Chinese dialysis patients. The mean level of hemoglobin in the present cohort was below 120 g/L, an optimal target recommended by KDOQI [20].

In the multivariate logistic regression analysis, the risk factors for cardiovascular morbidity in this cohort included old age, smoking, hypertension, hypertriglyceridemia, low level of hemoglobin, and elevated levels of C-reactive protein. It is interesting to note that the risk factors of CVD had different pattern between PD and HD patients. Metabolic disturbance-related risk factors, such as hyperglycemia, hypertriglyceridemia and hypoalbuminemia, were independently associated with cardiovascular morbidity only in PD patients. It has been shown that high glucose load and the presence of glucose degradation in conventional PD solution may lead to accumulation of advanced glycation end products which has been implicated in the development of CVD [17,18].

In the present study, the dialysis modality was not randomly selected and was affected by economic and social status as well as access to medical center. To confirm the impact of dialysis modality on CVD, a randomized control study would be needed. To reduce case mixed-related bias, this study included patients from nine of the largest dialysis facilities in China. Therefore, the results may only reflect the characteristic of dialysis population in academic and central dialysis facilities in China.

## Conclusion

We demonstrated that cardiovascular morbidity during chronic dialysis was higher than expected in a Chinese population. The cardiovascular morbidity was more prevalent in PD than HD patients among those over 50 years of age and those receiving dialysis for 3 years or more. The risk factors for CVD showed different pattern between PD and HD patients. Metabolic disturbance-related risk factors, such as hyperglycemia, hypertriglyceridemia and hypoalbuminemia, were independently associated with cardiovascular morbidity only in PD patients. Since CVD remains the leading cause of death in dialysis population worldwide, understanding the impact of dialysis modality on CVD in different population would be an important step for prevention and treatment.

## Methods

### Study population

The study cohort consisted of chronic patients from the hospital database of 9 of the largest dialysis facilities (the number of patients on HD ≥200 or PD ≥100) in 6 cities around China (Beijing, Shanghai, Guangzhou, Hangzhou, Wuhan, and Xian). The inclusion period for the present study was between January 1, 2005, and December 1, 2010. Among the cohort, 5.6% (HD, n = 125; PD, n = 8) of patients had been on chronic dialysis before transferred to the selected facility. Appropriate approval was obtained from the local ethics committee.

All adult (older than 18 years) patients with ESRD undergoing dialysis for at least 3 months in the selected facilities were enrolled. The patients who had clinically diagnosed ischemic heart disease, heart failure, stroke, and peripheral vascular disease at initiation of dialysis were excluded. Ischemic heart disease was defined as the presence of myocardial infarction, coronary revascularization procedures, angina, and ischemia on electrocardiogram or other diagnostic tests [5]. The diagnosis of heart failure was based on an ejection fraction below 40% or New York Heart Association Criteria grade 3 or more [21]. Peripheral vascular disease was defined as the presence of amputation of digits or extremities secondary to vascular disease, peripheral arterial bypass or angioplasty, intermittent claudication, recurrent cellulitis secondary to vascular disease [22].

A total of 2,388 (1,775 on HD and 613 on PD) patients were analyzed.

### Data collection

All data were collected at enrollment on the bases of review of medical records. All records were abstracted by two experienced dialysis research nurses. The following data were collected: demographic data, underlying renal diseases, medication records, dialysis modality, dialysis program, and cardiovascular morbidity which was defined as the presence of clinically diagnosed ischemic heart disease, heart failure, peripheral vascular disease, and/or stroke after initiation of dialysis.

Hematology and biochemistry measurements, time-averaged over the latest 3 months, were recorded. Fasting venous serum specimens were collected before dialysis session in HD patients or at the clinical visit in PD patients. Blood test was performed by the clinical laboratories in individual dialysis facilities.

Blood pressure measurement was taken before each of the three HD sessions or each of the three PD visits, all after 10 minutes of rest in a supine decubitus position.

### Dialysis regimens

Patients maintained on HD were dialyzed twice or thrice weekly with low-flux polysulphone or polyacrylamide dialyzer, either 1.5 or 1.7 m^2^ (Fresenius, Germany; Gambro, Sweden; Nipro, Japan; B. Braun, Germany; Langsheng, China). All treatments were of 4-hour to 5-hour duration with conventional glucose-free, bicarbonate-based dialysate containing 1.25 mM-1.5 mM calcium, 2.0 mM potassium, and 138 mM sodium. Dialysate flow was 500 ml/min.

All PD patients used lactate-buffered, 1.36% to 3.86% glucose-containing solutions (Baxter) as prescribed for routine clinical care. Patients were followed up every 1 ~ 3 months in each center.

### Statistical analyses

Statistical analyses were performed with SPSS 13.0 for Windows®. Descriptive statistics that used means, medians, proportions, SE, and confidence intervals were performed on all variables where appropriate. The continuous data were presented as the mean ± standard deviation or the median and interquartile ranges where appropriate. Categorical variables are presented as percentages. We compared two groups using the Student’s *t* test or the Mann–Whitney test where appropriate. The Pearson *χ*^2^ test and the Kruskal-Wallis test were applied for analysis of nominal and ordinal variables respectively. The odd ratios describing the association of selected risk factors with CVD were obtained by the univariable and multivariable logistic regression analysis, respectively.

## Abbreviation

CVD: Cardiovascular disease; HD: Hemodialysis; PD: Peritoneal dialysis; CCSD: China cooperate study on dialysis.

## Competing interests

All the authors declared no competing interests.

## Authors' contributions

FFH formed the concept and revised the manuscript; JPJ and SJW performed the experiment and measurements; PYC, DX, and XX analyzed the data; JHC, XQY, CLM, FX, WS, XSL, SRS, JL, PZ, YXZ, YMZ, XLL, ZMZ, QZL, YY enrolled the patients; QGZ drafted the manuscript. 

All authors read and approved the final manuscript.

## Pre-publication history

The pre-publication history for this paper can be accessed here:

http://www.biomedcentral.com/1471-2369/13/94/prepub
